# Circularly polarized light emission and detection by chiral inorganic semiconductors

**DOI:** 10.1007/s12200-024-00120-8

**Published:** 2024-05-31

**Authors:** Zha Li, Wancai Li, Dehui Li, Wei Tang, Huageng Liang, Huaibing Song, Chao Chen, Liang Gao, Jiang Tang

**Affiliations:** 1 Wuhan National Laboratory for Optoelectronics, Huazhong University of Science and Technology, Wuhan, 430074 China; 2 School of Optical and Electronic Information, Huazhong University of Science and Technology, Wuhan, 430074 China; 3 International Health Care Center, National Center for Global Health and Medicine, Tokyo, 162-8655 Japan; 4 Hepato-Biliary-Pancreatic Surgery Division, Department of Surgery, The University of Tokyo Hospital, Tokyo, 113-8655 Japan; 5 Department of Urology, Union Hospital, Tongji Medical College, Huazhong University of Science and Technology, Wuhan, 430074 China; 6 Faculty of Materials Science and Chemistry, China University of Geosciences, Wuhan, 430074 China

**Keywords:** High dissymmetric factor, Circularly polarized light emission, Semiconductor, Hard template, Chirality

## Abstract

Chiral inorganic semiconductors with high dissymmetric factor are highly desirable, but it is generally difficult to induce chiral structure in inorganic semiconductors because of their structure rigidity and symmetry. In this study, we introduced chiral ZnO film as hard template to transfer chirality to CsPbBr_3_ film and PbS quantum dots (QDs) for circularly polarized light (CPL) emission and detection, respectively. The prepared CsPbBr_3_/ZnO thin film exhibited CPL emission at 520 nm and the PbS QDs/ZnO film realized CPL detection at 780 nm, featuring high dissymmetric factor up to around 0.4. The electron transition based mechanism is responsible for chirality transfer.

**Graphical Abstract:**

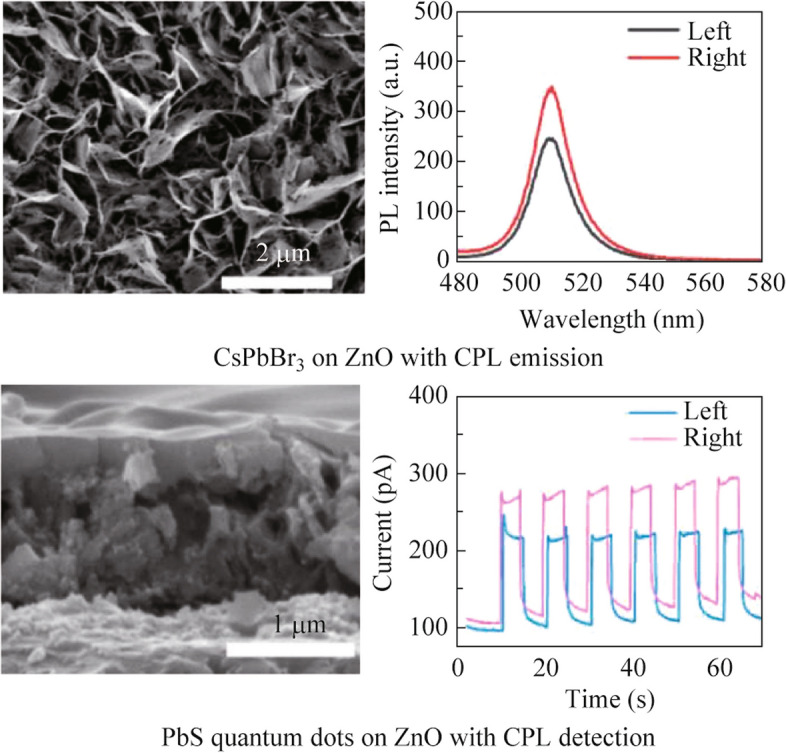

**Supplementary Information:**

The online version contains supplementary material available at 10.1007/s12200-024-00120-8.

## Introduction

Circularly polarized light (CPL) intrigues increasing attention for diverse applications, including 3D display, sensor, information storage, and etc. [1–4]. The CPL can be obtained by optical waveplates, but the use of waveplates is not beneficial to integrated devices. Thus, the exploration of novel materials and structures with the ability of CPL emission and detection is significative. Chiral organic molecules have demonstrated superior performance of CPL emission and detection, while the organic molecules suffer from the instability and low dissymmetry factors [5]. Generally the chiral inorganics are highly stable, but rarely exhibit CPL emission with high high dissymmetric factor (*g*_lum_) value because of their structure rigidity and symmetry.

To obtain chirality, inorganics generally require the interaction with chiral organic ligands or matrix [6, 7]. For example, nano-size inorganics exhibit tortured lattice or overall shape induced by chiral ligands [8]. The dissymmetry of the tortured lattice or shape leads to the CPL emission or absorption, but suffers the low dissymmetry factor due to the limited size of the dissymmetric core. As reported, the inorganic nano-materials such as perovskite quantum dots (QDs), CdSe QDs or Ag clusters induced by chiral organic molecules showed a low *g*_lum_ value at only 10−3 magnitude in CPL emission and absorption [9–11]. Hybrid halide perovskites, emerging as the attractive semiconductors, achieved chirality with an advanced *g*_lum_ value of ~0.1 by introducing organic chiral components as A site [12, 13], but the *g*_lum_ value is still not insufficient to significantly differentiate the left-hand and right-hand CPL.

There is an alternative method for enlarging the chirality of inorganics by embedding the nano-materials into the matrix with twisted pattern. For example, carbon dots arranged in helical cellulose matrix can demonstrate visible CPL emission with a high *g*_lum_ value of 0.74 [14], much larger than that of organics or chiral ligand coated QDs. Because the cellulose matrix exhibits micro-meter scaled helical superstructure, much larger than the size of chiral QDs. However, the cellulose is organic material, insulating and unstable to heat and mechanical force, which is unfavorable for device fabrication. Nano-materials of semiconductors can be assembled in twisted structure to obtain high chirality leading by chiral organic ligands [15]. Thus, the large chirality mainly rely on the organic ligands or matrix, and pure inorganic counterparts are still challenging. Recently, through calcination, pure chiral inorganics could be obtained with outstanding chirality due to their chiral structure, with the higher stability than the organics [16–18]. There is also some other chiral inorganics fabricated by using porous silica as template [19]. However, these chiral inorganics were all metal oxides and only presented CPL absorption or CD signals, no CPL emission. We proposed to use these chiral hard templates to transfer the chirality to other inorganic semiconductors for CPL emission and detection.

Metal oxides are commonly used inorganics in optoelectronic device and, by our literature review, only 4 metal oxides, including ZnO, CuO, SnO_2_ and TiO_2_, were reported as chiral films [16, 17, 20, 21]. Among them, the chiral ZnO film possesses the highest dissymmetry factor with the lowest cost [16]. Here, we chose the chiral ZnO film as template to introduce CPL emission from CsPbBr_3_ film and to enable CPL detection by PbS QDs. The precursor solution of CsPbBr_3_ was simply spin-coated and annealed on the chiral ZnO template. The CsPbBr_3_ layer succeeded the chiral structure directly from the ZnO template and exhibited strong CPL emission with a *g*_lum_ value of 0.41. This method is also available for other inorganic materials like PbS QDs, whose precursor solution was also spin-coated on the ZnO template with circular dichroism (CD signal at absorption range. The PbS QDs/ZnO film was assembled into photodetector and showed the ability of distinguishing left-/right-handed CPL at 780 nm with gdetect value around 0.4. Our method is universal for various semiconductors, which may open the gate to develop CPL emission and detection devices.

## Results and discussion

**Fig. 1:**
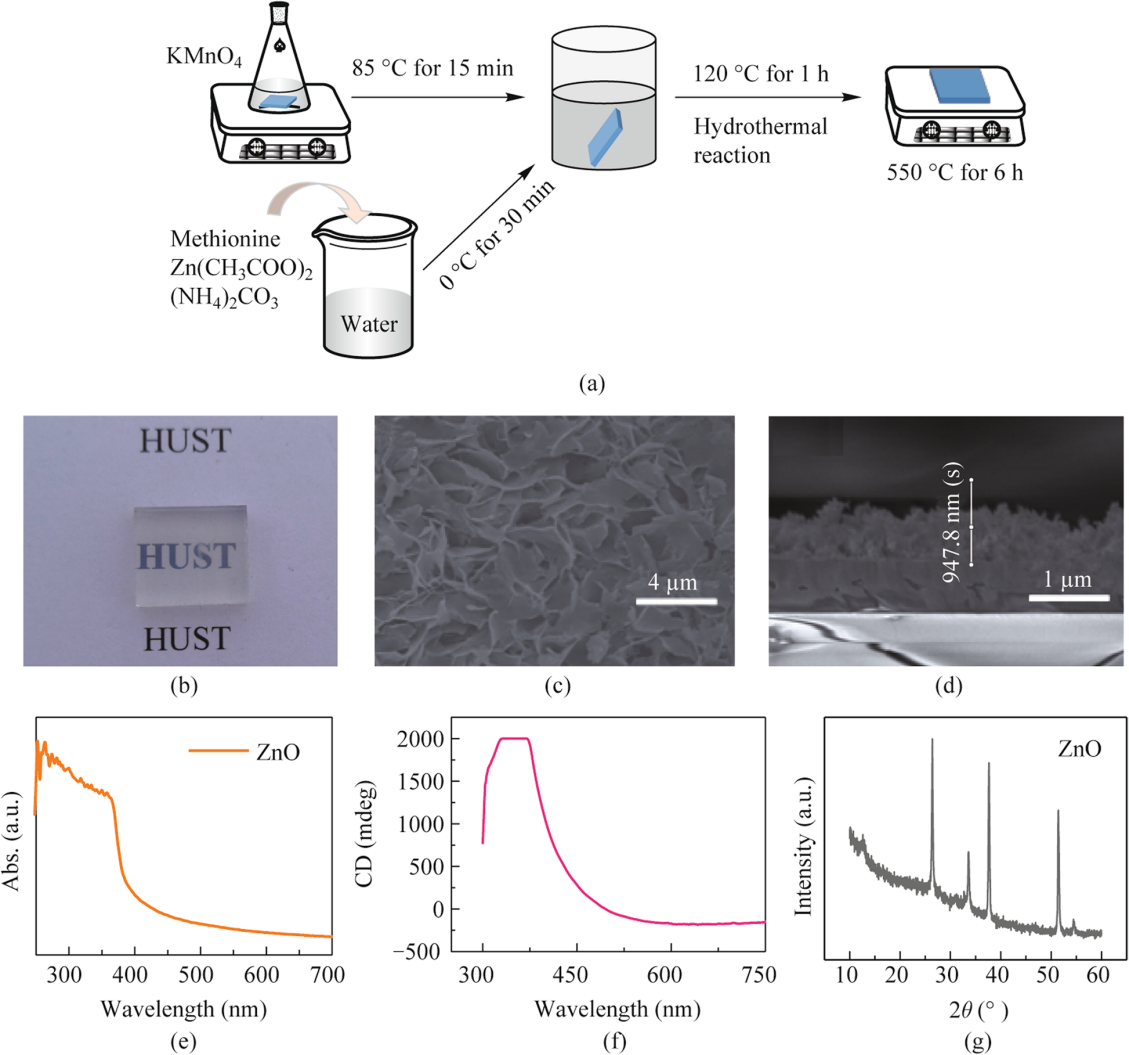
**a** Synthesis of chiral porous ZnO film on substrate. **b** Photo-graph of the obtained ZnO film on substrate. **c** Surface and **d** cross-section images of ZnO by SEM. **e** UV-Vis absorption and **f** CD spectrum of ZnO film. **g** XRD pattern of ZnO film

We synthesized chiral ZnO film on FTO and silica substrates as the reported procedure [16]. As illustrated in Fig. 1a, zinc acetate was used as Zn source and the natural chiral amino acid was used as symmetric-breaking agent to direct the chiral structure. After calcination at 600 °C for 6 h, the ZnO film looks semi-transparent (Fig. 1b) and exhibits the helical and porous structure as shown in SEM images (Figs. 1c and d). Regarding to the report, the hierarchy structure of ZnO leads to high optical activity and outstanding circular dichroism (CD) spectrum, which comes from the twisted crystalline structure [16]. The absorption spectrum and the CD spectrum of the obtained ZnO films are presented in Figs. 1e and f, showing CD over 2000 mdeg from 350 to 400 nm consistent with the literature. The X-ray diffraction (XRD) pattern indicates the typical ZnO crystalline peaks in Fig. 1g.

**Fig. 2:**
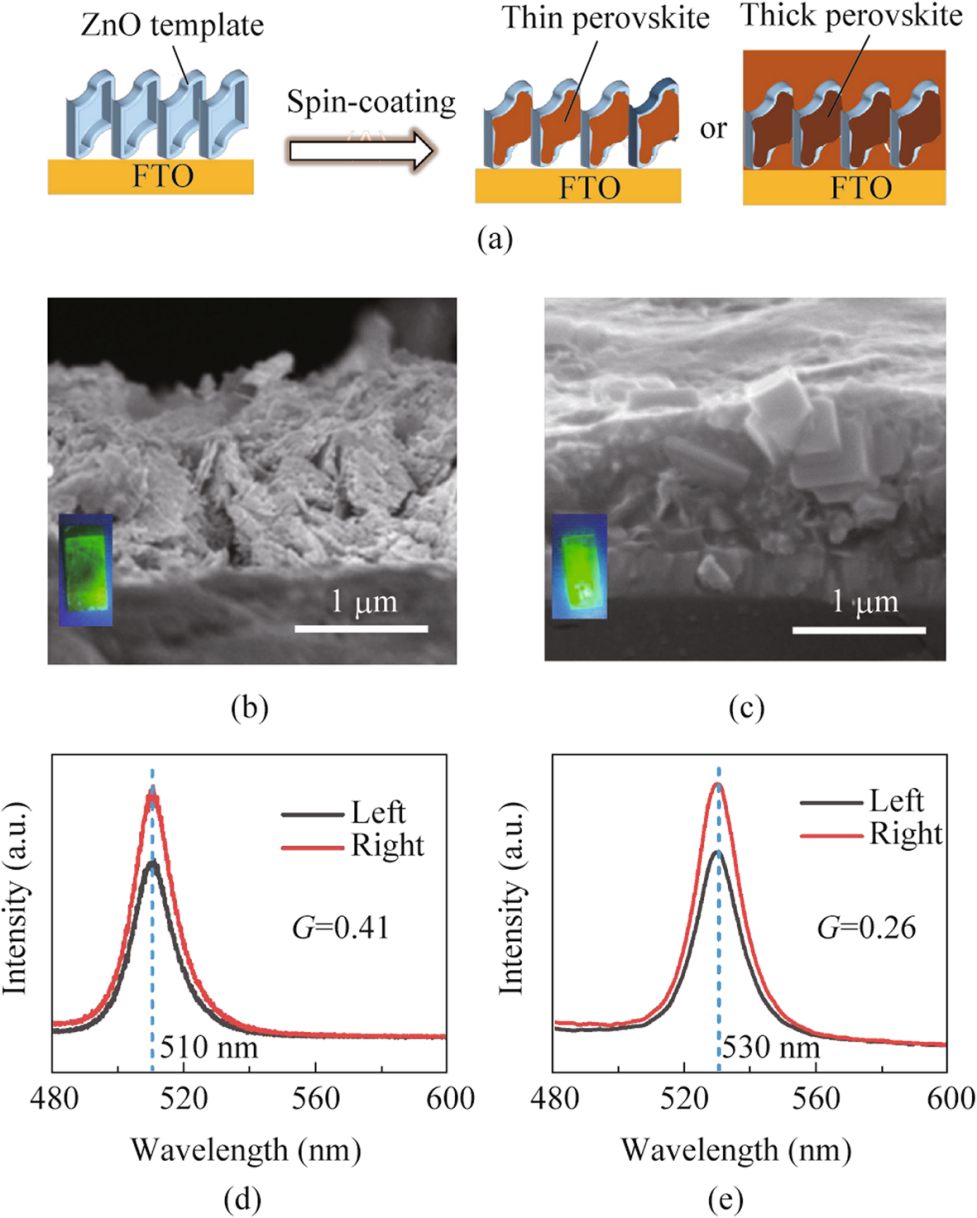
**a** Scheme of spin-coating perovskite on porous ZnO film. The porous structure is imprinted by thin or thick perovskite. Cross-section scanning electron microscope (SEM) images of **b** thin and **c** thick perovskite film on porous ZnO. Insert, the perovskite/ZnO under UV-irradiation. Left-hand and right-hand CPL spectra of **d** thin and **e** thick perovskite film on the chiral ZnO film

We dropped the CsPbBr_3_ precursor solution onto the ZnO film for 5 s incubation before the spin-coating. The morphology of ZnO is imprinted onto the CsPbBr_3_ layer with or without excessive reagents remaining as shown in Fig. 2a. The high-speed (8000 r/min) spin-coating could remove the excessive reagents and the CsPbBr_3_ that attached to the surface of the ZnO pores as a thin layer. The thin CsPbBr_3_ inherits the geometric shape of ZnO, obtaining the twisted crystalline structure and demonstrating the chirality (Figs. 2b, S1a and b). If spin-coating at slow speed (3000 r/min), the thick CsPbBr_3_ covers all over the ZnO structure (Fig. 2c). The *g*_lum_ value is defined by Eq. (1).

1 $${g}_{\text{lum}}=\frac{2\left|{\text{PL}}_{\text{r}} -{\text{ PL}}_{\text{l}}\right|}{\left|{\text{PL}}_{\text{r}} +{\text{ PL}}_{\text{l}}\right|},$$

where PL_r_ is right-hand photoluminescence (PL) and PL_l_ is left-hand PL. The thin CsPbBr_3_ exhibits the CPL emission at 510 nm with *g*_lum_ value around 0.41 (Fig. 2d), while the thick CsPbBr_3_ exhibits the CPL emission at 530 nm with decreased *g*_lum_ value around 0.26 (Fig. 2e). In the thick film, the excessive CsPbBr_3_ is achiral and thus only contributes to the non-handed PL, which decreases the *g*_lum_ factor.

The PL of ZnO is too low compared with the PL of CsPbBr_3_/ZnO as shown in Fig. S2. The chiral CsPbBr_3_/ZnO film exhibits the CPL emission at around 520 nm, attributed to the electron transition from conduction band to valence band of CsPbBr_3_ [19]. Referring to the chiral inorganics like TiO_2_, ZnO and CuO, the optical activity of CsPbBr_3_ is supposed to be the electronic transition based optical activity (ETOA) [16, 17, 22]. As the CsPbBr_3_ film succeeds to the structure from the chiral ZnO film, the CsPbBr_3_ film demonstrates chiral structure, offerring the anisotropic environment for the electron transition. The Coulomb interaction under the anistropic field impacts the electron transition of CsPbBr_3_ from the valence band to the conduction band and the reverse process.

According to the previous report, the hierarchical structure of the ZnO substrate enabled the high chirlity, including three levels: the primary helical ZnO crystalline structures within the nanoplates, the secondary helical structure of the stacks of the nanoplates, the tertiary assemblies by several stacks. To succeed the chirality of the ZnO substrate, it is plausible that the CsPbBr_3_ film exists in the rather limited inter-space between the ZnO nanoplates to succeed the similar hierarchical structure, meanwhile CsPbBr_3_ film possesses rather small thickness.

To further investigate the ETOA mechanism, we modified the coating process. We span the ZnO film at high speed first and then dropped the CsPbBr_3_ solution onto the furface of the ZnO film. Thus, the resulted morphology is the smooth CsPbBr_3_ layer lying on the ZnO film as indicated in SEM image (Fig. S3). The reason is that the pore size of the ZnO film is small and the surface tension delays the CsPbBr_3_ solution entering the pores. During the spin-coating process, the CsPbBr_3_ solution directly crystallizes over the pores and forms smooth CsPbBr_3_ film. If the scattering or reflection mechanism works, the chiral ZnO film should have worked as chiral filter and still lead to CPL emission. But the result did not show any CPL emission from CsPbBr_3_ film, denying this mechanism and favoring the electronic transition-based mechanism.

In the thin CsPbBr_3_ film case, the CsPbBr_3_ precursor solution enters into the pores of ZnO film. By high speed spin-coating, the excessive solution was removed and only the small volume parts attached to the ZnO surface and grew into crystallites in the rather confined space nearby. The resulted CsPbBr_3_ crystallites are rather small, directly attach to ZnO surface and follow the helical spatial arrangement, leading to the chiral structure and optical activity. The CPL emission exhibits a little hypochromic shift, mostly likely due to the confined size of crystallites.

For the thick CsPbBr_3_ film, less spin-coating speed retained more precursor solution in the pores or over the ZnO film. More CsPbBr_3_ crystallites are supposed to form freely in the pores, or even over the whole ZnO film, without attaching to the ZnO surface nor spatial confinement. Since they do not attach to the ZnO surface tightly nor follow the chiral ZnO structure, these parts exhibit no contribution to the chirality and lead to decreased *g*_lum_ value. By experiment, when the thickness over 2 µm, no CPL emission occurs and *g*_lum_ value is zero (Fig. S4). Without the spatial confinement, the PL spectrum from thick CsPbBr_3_ exhibits the bathochromic shift, which also supports our hypothesis. In addition, the CD spectra detected from different sides and rotation angles do not show obvious difference, which is supposed to eliminate the influence of linearly birefringence and linearly dichroism [17] (Fig. S5).

**Fig. 3:**
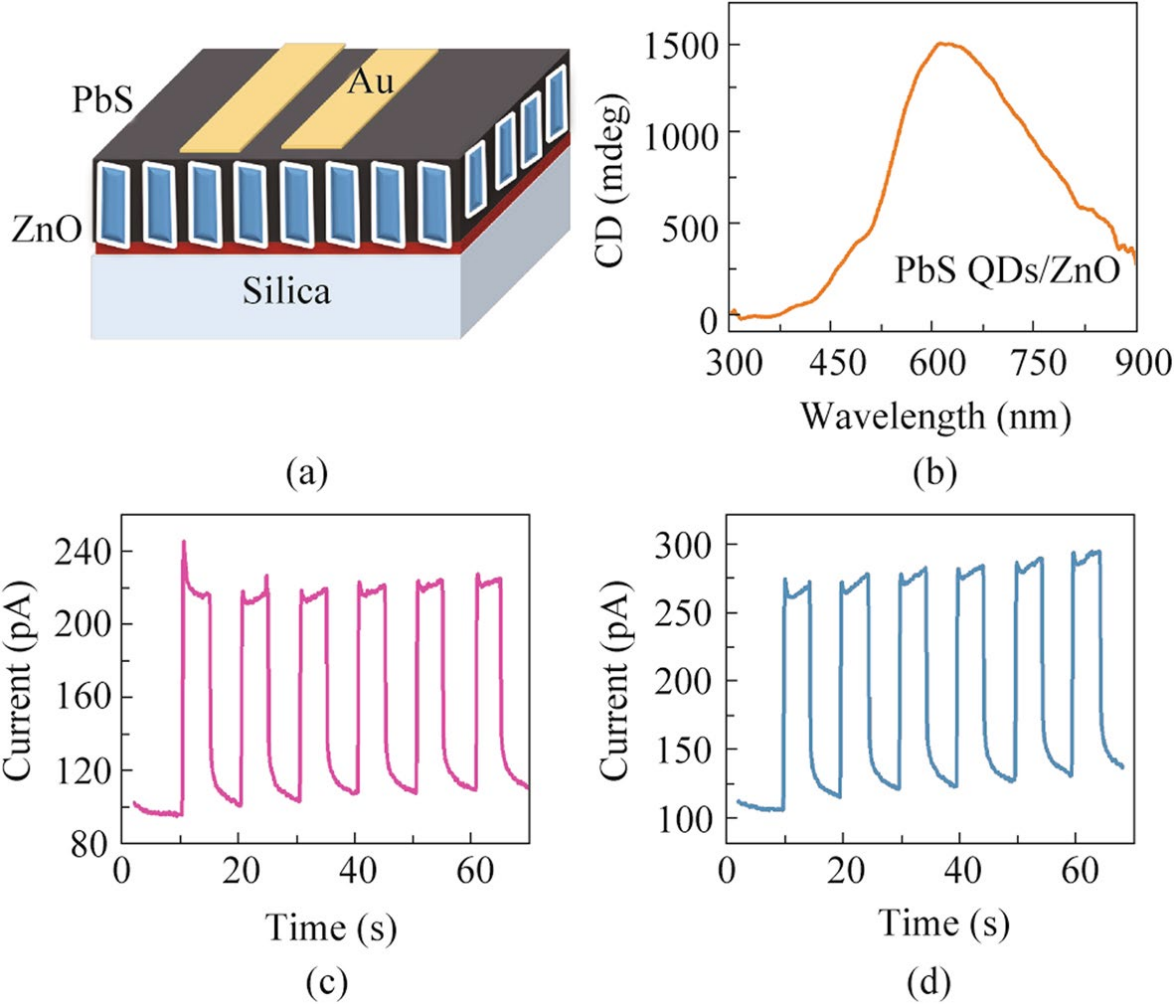
**a** Schematic illustration of photodetector of Au/PbS QDs/ZnO/silica. **b** The CD spectrum of the PbS QDs film on porous ZnO. The current–time curve of the photodetector under the **c** left-handed and **d** right-handed 780 nm irradiation with gdetect value calculated as 0.4

According to this electronic transition-based mechanism, other inorganics are also supposed to be applicable. We chose achiral PbS QDs as an active semiconductor on the chiral ZnO film for CPL detection. The PbS QDs solution was spin-coated on the ZnO film and the PbS QDs/ZnO thin film was obtained with the configuration in Fig. 3a. The PbS QDs/ZnO film exhibits the CD signals as shown in Fig. 3b, where the peak value is near to the absorption edge and attributed to the ETOA mechanism. The PbS QDs are aligned along the ZnO surface and form the helical structure film. In this film, the anisotropic electric field impact the electron transition process, including the exciton occurrence and separation, and the corresponding photo-current. We applied the chiral PbS QDs/ZnO film as a photodetector and measured by a home-made photo-detection system (Fig. S6). The 780 nm light-emitting diode was chosen as light source and modulated by the polarizer and 1/4 wave plate to generate the left- and right-handed CPL. The PbS QDs/ZnO film shows distinct response to left- and right-handed CPL irradiation with the same power density in Fig. S7 (*g*_detect_= 0.7). For CPL detection, the *g*_detect_ value is defined by Eq. (2) as follows:

2 $${g}_{\text{detect}}=\frac{2\left|{\text{PC}}_{\text{r}} -{\text{ PC}}_{\text{l}}\right|}{\left|{\text{PC}}_{\text{r}} +{\text{ PC}}_{\text{l}}\right|},$$

where PC_r_ and PC_l_ are the net photocurrents to right- and left-handed light, respectively. The response speed is rather slow, likely due to the interaction between ZnO and PbS QDs (Fig. S7). To enhance the response speed, we deposited an Al_2_O_3_ layer of 3 nm thickness on the ZnO film as insulator by atomic layer deposition (ALD) before spin-coating PbS QDs. The Al_2_O_3_ layer blocked the interaction between ZnO and PbS QDs, but retained the chiral structure of the ZnO film because of conformal coating of Al_2_O_3_. Consequently, the response speed is enhanced greatly and the CPL distinction remains significant with gdetect value around 0.4 as shown in Figs. 3c and d.

In summary, we have demonstrated a universal method to transfer chirality from chiral porous inorganic hard template to other achiral inorganic semiconductors. The resulted semiconductors succeed the chiral structure and high chiral activity based on electronic transition with the high dissymmetric value. In this study, we used the chiral ZnO film as hard template and coated CsPbBr_3_ and PbS QD as the active semiconductors. The chiral CsPbBr_3_ film exhibit CPL emission at 520 nm with *g*_lum_ up to 0.4. The PbS QDs film distinguish CPL at 780 nm with gdetect up to 0.4. We prove that the chiral porous hard template endows the chirality to other semiconductors for chiral sensitive applications. The ETOA mechanism is responsible for the transferred chirality and the wavelength is tunable referring to the band gap.

## Experimental section

### Reagents

Lead bromide (PbBr_2_, 98%), lead iodide (PbI_2_, 99%) and cesium bromide (CsBr, 99%) were purchased from Aladdin. DMF, zinc acetate dihydrate (Zn(OAC)_2_·2H_2_O), Methionine, (NH_4_)_2_CO_3_, KMnO_4_ and butanol were purchased from Sinopharm Chemical Reagent Co. Ltd. All reagents were directly used without any further purification.

### Fabrication of chiral porous ZnO film

FTO or silica substrate was sequentially rinsed by detergent solution, water, acetone and ethanol in ultrasonic bath for 10 min. Then the clean substrate was incubated in 20 mL 10 mmol/L KMnO_4_ solution/50 μL of butanol mixture at 85 °C for 15 min for activation. The resulted FTO was yellowish transparent. 2 mmol methionine and 3 mmol Zn(CH_3_COO)_2_·2H_2_O were mixed and dissolved in 25 mL water solution with stirring. 1 mmol (NH_4_)_2_CO_3_ was added and stirred in ice-water bath for 0.5 h. Then the activated substrate was placed in 25 mL Teflonlined autoclave with immersion into the suspension to react at 120 °C for 1 h. Then the substrate with ZnO film was washed by water and ethanol for several times. After dried in the air, the ZnO/substrate was followed by slow heating procedure of 6 h to 550 °C and calcination for 6 h.

### CsPbBr_3_ spin-coating

25 mmol/L CsBr and PbBr_2_ were dissolved in DMSO by long time stirring as CsPbBr_3_ precursor solution. The spin-coating speed was adapted from 3000 to 8000 r/min. Then, to enhance the brightness, we dropped 10 mg/mL MABr/isopropanol on the film and spin-coated at 5000 r/min for 30 s. The films were dried in air at 100 °C on the hot-plate.

### PbS QD synthesis

Following the previous protocol, 0.9 g PbO, 2.9 g oleic acid (OA) and 20 mL octadecene (ODE) were degassed at 85 °C with 6 h stirring. The Pb-source solution became colorless transparent. Then, 320 µL hexamethyldisilathiane (TMS) dissolved in 10 mL degassed ODE was quickly injected into the Pb-source solution at 85 °C and heated for 10 s. Then the heating mantle was directly removed and the mixture naturally cooled down to the room temperature. The PbS QDs were washed and purified by precipitation.

### Al_2_O_3_ atomic-layer-deposition (ALD)

The ALD is executed by ASM Pulsar2000™ ALD module to grow the Al_2_O_3_ layer. The Al_2_O_3_ was grown by alternating pulses of Al(CH_3_)_3_ and H_2_O with the carrier gas flow of nitrogen. By controlling the number of ALD cycles, the thicknesses 30 Å were set with the reaction temperature of 300 °C.

### CPL emission measurement

CPL emission measurement was carried out on a homemade Raman spectrometer system with a 405 nm linearly polarized laser light source at 0.1 μW. The CPL passed through a quarter-wave plate followed by a polarizer. The transmission was recorded by a CCD instrumentation (Symphony II, Horiba). The schema of the system was illustrated in the supporting information (Fig. S8).

### Characterization

The scanning electron microscopic measurement (SEM) was carried out by FEI Nova Nano 450 SEM. TEM observations were performed on a Tecnai G2 20U-TWIN machine at 300 kV. The XRD measurement was executed on Philips X pert pro MRD diffractometer with Cu Kα radiation. The CD spectrum were obtained using JASCO J-810 spectrophotometers.

### Supplementary Information

Supplementary Material 1.

## Data Availability

The data that support the findings of this study are available from the corresponding author, upon reasonable request.
