# Supplementary material for: Circularly polarized light emission and detection by chiral inorganic semiconductors
Source: Front Optoelectron. 2024 May 31;17(1):15. doi: 10.1007/s12200-024-00120-8 (PMC11143083; doi:10.1007/s12200-024-00120-8)
Supplement: Supplementary file 1 — Supplementary Material 1. [file 12200_2024_120_MOESM1_ESM.pdf]

Supporting Information

Circularly polarized light emission and detection by chiral inorganic semiconductors

Zha Li[1](✉), Wancai Li[2], Dehui Li[2], Wei Tang[3,4], Huageng Liang[5], Huaibing Song[6], Chao Chen[2], Liang Gao[1](✉), Jiang Tang[1,2]

[1] Wuhan National Laboratory for Optoelectronics, Huazhong University of Science and Technology, Wuhan 430074, China

[2] School of Optical and Electronic Information, Huazhong University of Science and Technology, Wuhan 430074, China

[3] International Health Care Center, National Center for Global Health and Medicine, Tokyo 162-8655, Japan

[4] Hepato-Biliary-Pancreatic Surgery Division, Department of Surgery, The University of Tokyo Hospital, Tokyo 113-8655, Japan

[5] Department of Urology, Union Hospital, Tongji Medical College, Huazhong University of Science and Technology, Wuhan 430074, China

[6] Faculty of Materials Science and Chemistry, China University of Geosciences, Wuhan 430074, China

Corresponding authors

highlight@hust.edu.cn (Liang Gao)

zhali@hust.edu.cn (Zha Li)

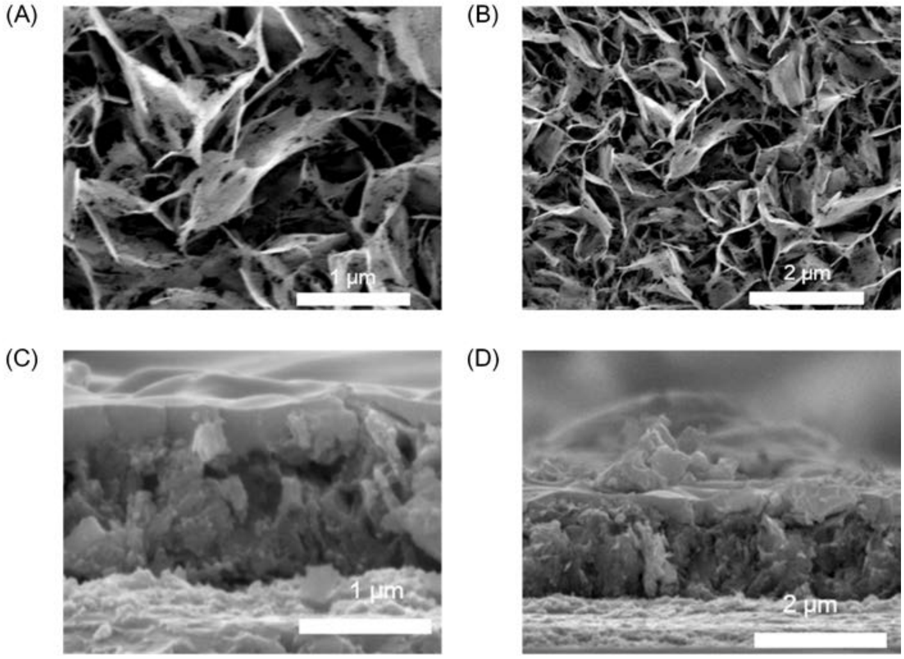

Figure S1. A) and B) The SEM images at varying magnifications of CsPbBr3/ZnO film. C) and D) The cross-section SEM images at varying magnifications of the PbS QDs/ZnO film in our study.

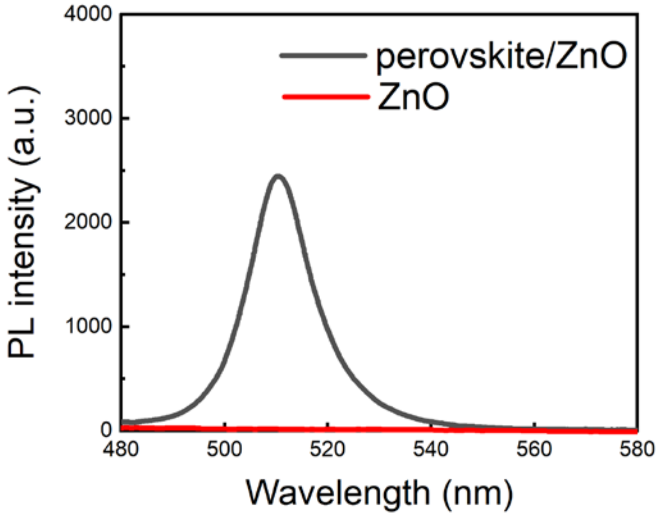

Figure S2. The PL spectra of ZnO film and perovskite $CsPbBr_3$/ZnO film.

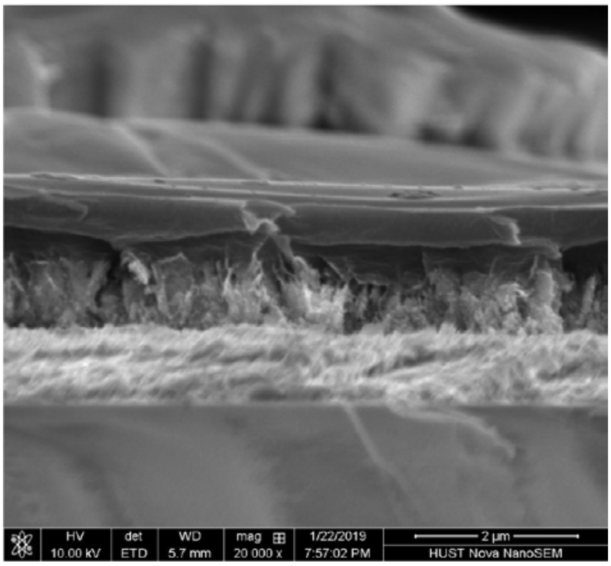

Figure S3. The cross-section SEM image for the $CsPbBr_3$/ZnO with the $CsPbBr_3$ layer lying on the porous ZnO layer and g value of zero for CPL emission.

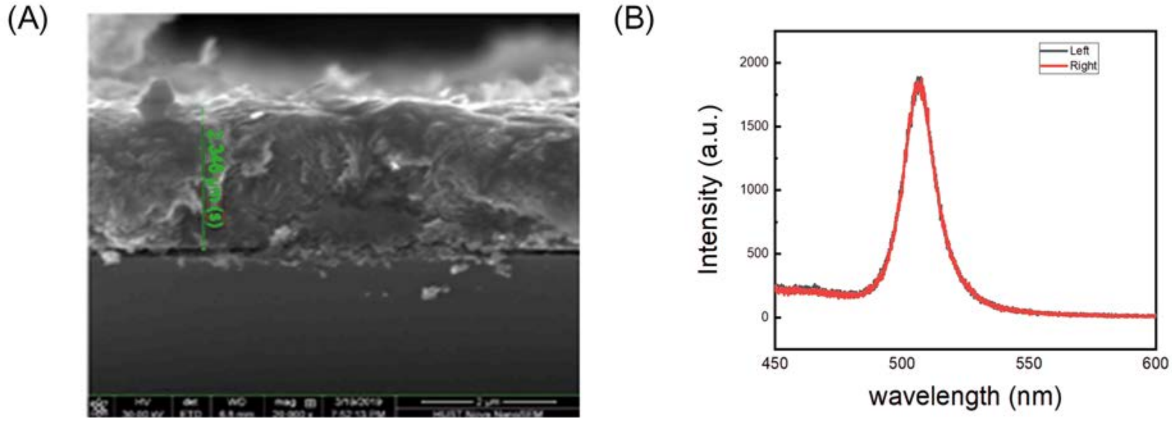

Figure S4. (A) The cross-section SEM image for the $CsPbBr_3$/ZnO with the $CsPbBr_3$ thickness around 2.3 μm. (B) The left-handed and right-handed PL spectrum of this $CsPbBr_3$/ZnO with the zero valued g_{lum}.

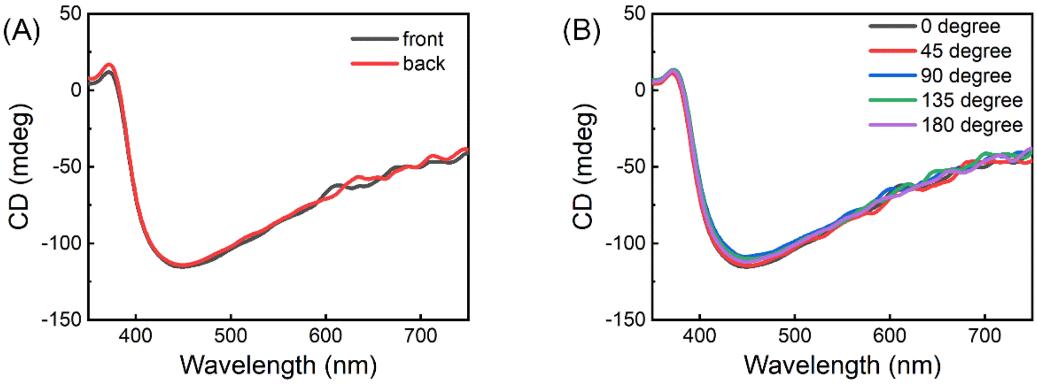

Figure S5. The CD spectra of the ZnO films by measuring (A) from front-side and back-side and (B) with different rotating angles from 0 degree to 180 degree.

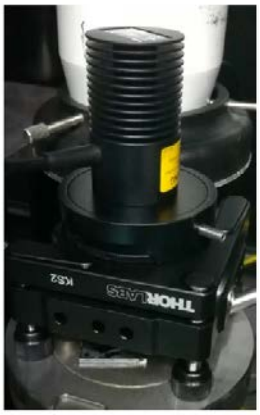

Figure S6. The home-made CPL detector system.

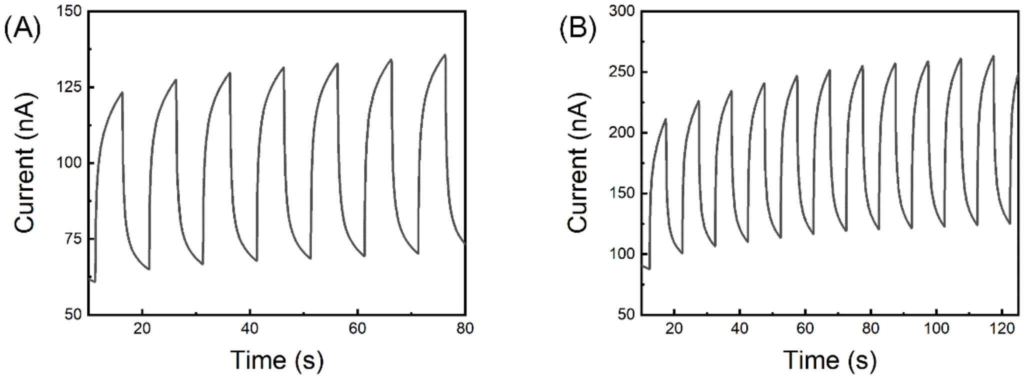

Figure S7. The current-time curve of PbS QDs/ZnO photodetector under the left-/right-handed CPL irradiation with the same power density.

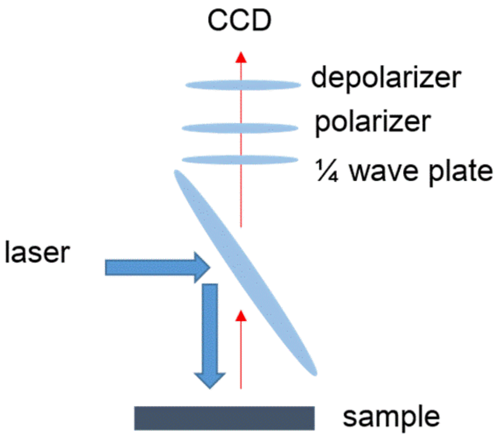

Figure S8. The schema of the home-made Raman spectrometer system with laser source for CPL emission detection.
